# Failure to Notify Infectious Disease

**Published:** 1910-06-11

**Authors:** 


### Medico-Legal Points.

### FAILURE TO NOTIFY INFECTIOUS DISEASE.

On October 21, before the Kingston County Bench, George Stead, of Sussex Road, New Malden, chartered accountant, was summoned for failing as head of the family, as soon as he became aware on or before July 29, 1909, to notify that his three children were suffering from an infectious disease—namely, scarlet fever. The proceedings were taken under the Infectious Diseases (Notification) Act, 1889, which Act is made applicable to the district by the Infectious Disease (Notification) Extension Act, 1899, which provides *inter alia*: "The head of the family to which such patient belongs . . . . shall as soon as he becomes aware that the patient is suffering from an infectious disease to which this Act applies, send notice thereof to the medical officer of health of the district.' The suspicions of the sanitary officials was first aroused in connection with an investigation made of a case of scarlet fever in the district and in the same locality. Owing to certain suspicions the sanitary inspector called at the defendant's house on July 29, when the wife of the defendant first denied that there was any illness whatever in the house, and upon calling her husband he ultimately admitted that his children were suffering from sore throats, followed by a rash which appeared over the body of each child. He treated the matter very lightly, and stated that he had not consulted a medical man. The inspector then asked to see the children, but he was only allowed to see one child, and then only at the door of the house. He suspected that the child was suffering from scarlet fever and told the defendant so, and advised him to consult a medical man; but this he refused to do. Then the inspector offered to bring a medical man, or the medical officer of health, to see the children at the Council's expense; but this was flatly refused. The inspector then consulted the medical officer of health, and they both decided that it would be best to call at the house in about fourteen days' time, when the peeling would be at its height provided that their suspicions were correct. On August 12 (about fourteen days after his first visit) the inspector again called at the defendant's house, accompanied by the medical officer of health, and saw the defendant's wife. After considerable persuasion they were admitted to the premises, and the medical officer of health found, after examining the three children, that they were in a peeling condition from scarlet fever. During this period, although the defendant had had his suspicions aroused by the sanitary inspector, and had been given printed instructions as to isolation, he had neither consulted a medical man nor made any attempt to isolate his children. The defence was that the defendant and his wife were absolutely unaware of the presence of scarlet fever in the house, and the sanitary inspector did not inform them of its presence upon the occasion of his visit. The Bench imposed the maximum penalty of £2, and three guineas was allowed as costs to the Council, while the Court costs also have to be borne by the defendant. The Chairman commented severely on the case, and asked wherein came the use of public authorities doing their utmost to eradicate infectious disease when an obstinate man like the defendant deliberately went and spread it about. The case ought to be a warning to people to act with more common sense.

